# Risks of hormonally active pharmaceuticals to amphibians: a growing concern regarding progestagens

**DOI:** 10.1098/rstb.2013.0577

**Published:** 2014-11-19

**Authors:** Moa Säfholm, Anton Ribbenstedt, Jerker Fick, Cecilia Berg

**Affiliations:** 1 Department of Organismal Biology, Environmental Toxicology, Uppsala University, Norbyvägen 18A, 75236 Uppsala, Sweden; 2 Department of Chemistry, Umeå University, KBC 6A, Linnaeus väg 6, 90187 Umeå, Sweden

**Keywords:** endocrine disruption, oogenesis, fertility

## Abstract

Most amphibians breed in water, including the terrestrial species, and may therefore be exposed to water-borne pharmaceuticals during critical phases of the reproductive cycle, i.e. sex differentiation and gamete maturation. The objectives of this paper were to (i) review available literature regarding adverse effects of hormonally active pharmaceuticals on amphibians, with special reference to environmentally relevant exposure levels and (ii) expand the knowledge on toxicity of progestagens in amphibians by determining effects of norethindrone (NET) and progesterone (P) exposure to 0, 1, 10 or 100 ng l^−1^ (nominal) on oogenesis in the test species *Xenopus tropicalis*. Very little information was found on toxicity of environmentally relevant concentrations of pharmaceuticals on amphibians. Research has shown that environmental concentrations (1.8 ng l^−1^) of the pharmaceutical oestrogen ethinylestradiol (EE_2_) cause developmental reproductive toxicity involving impaired spermatogenesis in frogs. Recently, it was found that the progestagen levonorgestrel (LNG) inhibited oogenesis in frogs by interrupting the formation of vitellogenic oocytes at an environmentally relevant concentration (1.3 ng l^−1^). Results from the present study revealed that 1 ng NET l^−1^ and 10 ng P l^−1^ caused reduced proportions of vitellogenic oocytes and increased proportions of previtellogenic oocytes compared with the controls, thereby indicating inhibited vitellogenesis. Hence, the available literature shows that the oestrogen EE_2_ and the progestagens LNG, NET and P impair reproductive functions in amphibians at environmentally relevant exposure concentrations. The progestagens are of particular concern given their prevalence, the range of compounds and that several of them (LNG, NET and P) share the same target (oogenesis) at environmental exposure concentrations, indicating a risk for adverse effects on fertility in exposed wild amphibians.

## 1. Introduction

Do pharmaceuticals pose a risk to amphibians? While it is established that pharmaceutical steroids in the aquatic environment present a risk to fish [1], the knowledge on potential risks to amphibians is much less developed [2]. The objectives of the present paper are (i) to review the scientific literature regarding adverse effects of environmentally relevant concentrations of hormonally active pharmaceuticals on amphibians and (ii) to contribute new knowledge on effects of the progestagens norethindrone (NET) and progesterone (P) on the female reproductive system in amphibians. The review focuses on risks of water-borne pharmaceuticals to amphibians; therefore, the compounds addressed are those that have been detected in surface water and for which there are available toxicity data in amphibians, i.e. steroidal hormones (oestrogens, androgens and progestagens), selective serotonin re-uptake inhibitors (SSRIs) and aromatase inhibitors. We focused primarily on toxicity data for environmentally relevant exposure levels when available.

## 2. Amphibian exposure scenarios

Amphibians inhabit a wide range of environments but a common feature is that most species, including the terrestrial ones, breed in water. Many amphibians are generalists and spawn in virtually all types of water bodies, including rivers, lakes, ditches and marine environments, whereas some species are specialized for breeding in, for example, ponds (e.g. *Rana sylvatica*) or rivers (e.g. *Rana boylii, Hyla rivularis*, *Ascaphus truei*) [3,4]. The larval period is a critical period for sex differentiation in many amphibians (including *Xenopus* and *Rana* species) and exposure during this life-stage to hormonally active chemicals can disrupt gonadal differentiation (reviewed in [5]). Another sensitive phase of the reproductive cycle is the maturation and release of gametes. During the breeding season, which can last for several months, the adult frogs may be exposed to water-borne chemicals at critical phases of egg and sperm maturation [3].

Amphibians may be exposed to pharmaceuticals via the water during the breeding period and the larval stages in waterways polluted by municipal wastewater or emissions from drug manufacturing sites, as well as in agricultural areas irrigated with wastewater or where domestic stock is kept [6–9]. A number of studies report the presence of hormonally active pharmaceuticals including steroid hormones, aromatase inhibitors and SSRIs in typical breeding habitats for amphibians, including lakes, rivers and streams [10–16].

### (a) Types and levels of hormonally active pharmaceuticals in the environment

Natural and synthetic steroid hormones including oestrogens, androgens and progestagens (here defined as natural or synthetic progesterone) are widely used in human and veterinary medicine, in contraceptives or other hormonal therapies. Whereas there are quite a few data on environmental concentrations of oestrogens, less information is available for the environmental concentrations of pharmaceutical androgens and progestagens (reviewed in [17]). Reported concentrations of oestrogens (including EE_2_) and progestagens (e.g. levonorgestrel (LNG), NET and P) in surface waters (lakes, rivers, streams) are often in the 1–10 ng l^−1^ range [9–12,14,16] or below (reviewed in [17]). Testosterone has been detected at concentrations up to 6 ng l^−1^ in ground water [14] and methyldihydrotestosterone (MDHT), a non-aromatizable anabolic steroid, has been reported to be present in a river in the tens of ng l^−1^ range [18]. A recent study of river water downstream of a pharmaceutical industry effluent discharge in France revealed that progesterone receptor agonists were among the most abundant types of hormonally active pharmaceuticals, as determined by chemical analysis and *in vitro* bioassays [9].

Pharmaceuticals that inhibit CYP 19 (aromatase), the enzyme that converts androgens into oestrogens, have potential to impair reproductive development and function (reviewed in [19]). The imidazole clotrimazole is a widely used antifungal agent (in e.g. anti-dandruff shampoos) which is released into the aquatic environment from wastewater treatment works and hospitals [20,21]. In a survey of five UK rivers, clotrimazole was detected in 100% of the samples at concentrations ranging from 6 to 34 ng l^−1^ with a median of 21 ng l^−1^ [20].

SSRIs act by increasing extracellular serotonin levels and serotonin neurotransmission and are commonly prescribed for depression, compulsive behaviours and eating disorders. A number of SSRIs, including fluoxetine, sertraline and citalopram, have been detected in the aquatic environment at concentrations up to 0.1 µg l^−1^ downstream of wastewater treatment plants [10,22,23]. Fluoxetine was shown to have oestrogenic activity *in vitro* and in uterotropic assay in rats [24]. In fish studies, exposure to 0.1–0.5 µg fluoxetine l^−1^ has been shown to increase the plasma oestradiol levels, indicating endocrine effects [25,26].

## 3. Review of effects of hormonally active pharmaceuticals in amphibians

It is well established that exposure to elevated concentrations (in the μg l^−1^ range) of oestrogens, androgens and progestagens during the larval period can disrupt gonadal differentiation resulting in skewed sex ratios at metamorphosis (reviewed in [5]). There is, however, a very limited number of studies that have investigated adverse effects of environmentally relevant concentrations of steroidal hormones on amphibians.

### (a) Oestrogens

Studies in a number of amphibian species have shown that larval exposure to EE_2_ concentrations in the μg l^−1^ range can induce female-biased sex ratios indicating male-to-female sex reversal [27,28]. A life-cycle study on *Xenopus tropicalis* demonstrated that larval exposure to EE_2_ at concentrations in the low ng l^−1^ range did not only induce a permanently skewed sex ratio towards females, but also impaired spermatogenesis and reduced fertility in the males as they reached sexual maturity [29]. The frogs were exposed to 1.8, 18 and 180 ng EE_2_ l^−1^ for seven weeks from shortly after hatching until metamorphosis, after which they were kept in clean water. Eight months after metamorphosis, there was an increasing proportion of phenotypic females (as determined by histological gonadal sex) with increasing exposure level compared with the control group, indicating male-to-female sex reversal. The fertilization rate of the males that were not sex-reversed (i.e. 14 males exposed to 1.8 ng l^−1^ and 1 male exposed to 18 ng l^−1^) was reduced to about 50% of that of the control males when mated with unexposed females. The males exposed to 1.8 ng EE_2_ l^−1^ had fewer mature spermatozoa in the lumen of the seminiferous tubules compared with the control group suggesting that a reduced production of mature spermatozoa had a role in the reduced fertility rate. A significant proportion of the adult EE_2_ exposed phenotypic females lacked oviducts, making them sterile [29]. It was concluded that testicular development and differentiation of the Müllerian ducts (embryonic precursors of the female reproductive tract in vertebrates except teleost fish) are sensitive targets for oestrogen exposure in amphibians. These findings were the first to demonstrate reproductive toxicity following exposure to environmentally realistic EE_2_ concentrations. The sensitivity to EE_2_ of the model species *X. tropicalis* was demonstrated to be comparable to that of the temperate, terrestrial species the common frog, *Rana temporaria* [30]. As the exposure scenario with respect to the level, route and duration was ecologically relevant and the endpoint studied (i.e. fertilization rate) has relevance to population dynamics this study indicates that EE_2_ might pose a threat to wild frog populations.

Little is known regarding the mechanisms underlying the developmental reproductive effects of EE_2_ in amphibians. However, an RNA expression analysis of genetically male *Xenpus laevis* tadpoles exposed to 840 ng EE_2_ l^−1^ showed that three transcripts involved in development of the testis and spermatogenesis (i.e. celf1, dmrt1b and gtsf1) were downregulated compared with the controls [31].

### (b) Androgens

Exposure to 30 ng 17α-MDHT l^−1^ (nominal concentration) enhanced the advertisement calling in male *X. laevis* frogs, indicating an increase in sexual arousal [32]. Males (10 per group) were individually exposed to 0.03, 3 and 30 µg MDHT l^−1^ (nominal concentrations) for 96 h and calling behaviour was recorded each night. The advertisement calling frequency was increased from about 85% in the controls to 95% and above in all MDHT treatment groups. The mechanism of action for this effect was suggested to be an interaction with androgen receptors either locally in the larynx or in the vocal pathway in the central nervous system [32]. Whether or not increased calling behaviour can have adverse effects on the individual or population remains to be determined.

### (c) Progestagens

Levonorgestrel has been shown to act as a reproductive toxicant in amphibians, both after larval and adult exposure [33,34]. A life-cycle study on *X. tropicalis* revealed that female frogs exposed to 156 ng l^−1^ during the tadpole stage, and thereafter reared in clean water until they reached sexual maturity, were sterile upon mating with unexposed males [33]. The LNG-exposed females completely lacked oviducts and the percentage of maturing oocytes (i.e. from the first follicular stage up to the mature oocyte) was drastically decreased from about 55% in the controls to about 5% in the exposed females, as determined by a histological evaluation of the ovaries. These results indicate that larval LNG exposure targeted both Müllerian duct differentiation and oogenesis resulting in permanently impaired fertility. No effects of larval exposure to 19 or 156 ng LNG l^−1^ on the sex ratio or on ovarian histology were detected at metamorphosis [33]. The implication of these findings is that a full life-cycle test seems necessary to disclose the severe consequences of developmental exposure to this type of compound. No effects on testicular development, sperm count or fertility were observed in adult male *X. tropicalis* exposed to 19 or 156 ng LNG l^−1^ during the larval stage suggesting that females are more susceptible than males to developmental toxicity of progestagens in amphibians [33].

In adult *X. tropicalis* females, exposure to LNG for four weeks at concentrations as low as 1.3 ng l^−1^ interrupted oogenesis as determined by a histological evaluation of the ovaries [34]. In females exposed to 1.3 ng LNG l^−1^ the proportion of vitellogenic oocytes was reduced by 63% compared with the control and the proportion of previtellogenic oocytes was increased by 32% compared with the control [34]. The results imply that oogenesis, and in particular vitellogenesis, is targeted by LNG at low ng l^−1^ concentrations in adult female amphibians. In a study on *X. tropicalis* males (*n* = 7–12 per exposure group) exposed to 3, 22 or 187 ng LNG l^−1^ (measured levels) for four weeks, no effects on testicular histology or sperm quality were detected (M Säfholm, J Fick, C Berg 2014, unpublished results). In male *X. laevis* frogs, exposure for 96 h to LNG was shown to increase advertisement calling at exposure levels of 30 ng l^−1^ and higher [35]. It remains to be determined whether calling behaviour is also affected at lower, more environmentally relevant exposure levels. The susceptibility of amphibians to reproductive toxicity caused by progestagens other than LNG present in the environment is not known.

### (d) Selective serotonin re-uptake inhibitors

To determine the effects of exposure to fluoxetine on amphibian development, *X. tropicalis* larvae were chronically exposed for eight weeks via the ambient water to 0.6 or 9.6 µg l^−1^ fluoxetine from hatching until metamorphosis [36]. The animals exposed to 9.6 µg fluoxetine l^−1^ had an increased concentration of the serotonin metabolite 5-hydroxyindoleacetic acid in the hypothalamus, compared with controls. None of the exposure levels caused any significant changes in time to metamorphosis, thyroid histology, gonadal sex differentiation or aromatase activity in brain and gonads indicating that the effect on the serotonergic system in the hypothalamus was specific [36]. In another study, *X. laevis* tadpoles were exposed until metamorphosis (for 10 weeks) to fluoxetine or the SSRI sertraline at 0.1, 1.0 and 10 μg l^−1^ to determine effects on growth and development. Tadpoles exposed to fluoxetine (10 µg l^−1^) or sertraline (0.1, 1 and 10 µg l^−1^) exhibited reduced weight at metamorphosis and it was concluded that only sertraline was capable of affecting larval development at environmentally relevant concentrations [37]. This warrants further research into the consequences of developmental exposures to SSRIs in amphibians.

### (e) Aromatase inhibitors

Larval exposure to aromatase inhibitors such as fadrozole and flavone has been shown to disrupt ovarian differentiation resulting in the development of testicles or intersexed gonads in amphibian females [28,38,39]. While these studies demonstrate the susceptibility of ovarian differentiation to aromatase inhibition, very little is known about the effects of exposure to aromatase-inhibiting pharmaceuticals that occur in the aquatic environment. A study on *X. tropicalis* evaluated the effects of seven weeks’ exposure to 2, 14 and 129 µg clotrimazole l^−1^ on gonadal differentiation and aromatase activity in brain and gonads during gonadal differentiation [40]. No effects of clotrimazole on gonadal differentiation were detected, whereas gonadal aromatase activity was increased in both sexes at exposure levels greater than or equal to 14 µg l^−1^ [40]. To the best of our knowledge, no data on adverse effects of environmentally relevant concentrations of clotrimazole or other pharmaceutical aromatase inhibitors on amphibians have been reported.

## 4. Experimental assessment of the effects of the progestagens norethindrone and progesterone on oogenesis

Considering that several progestagens (including NET and P) are present in the environment, information on their effects and potencies is needed in order to understand the risks this group of substances present to exposed wild amphibians. With the aim to determine the adverse effects of NET and P on oogenesis, adult *X. tropicalis* females were exposed via the ambient water for 28 days to 1, 10 or 100 ng l^−1^ (nominal) of the test compounds after which the full cycle of oogenesis was analysed histologically.

### (a) Methods

#### (i) Experimental set-up

Two experiments were carried out as follows. Sexually mature female *X. tropicalis* (European Xenopus Resource Centre, Portsmouth, UK, experiment 1, or Xenopus One, Dexter, US, experiment 2) were exposed individually to NET (purity > 98%, CAS: 68-22-4, Sigma-Aldrich, Steinheim, Germany) or P (purity > 97%, CAS: 57-83-0, Sigma-Aldrich, Steinheim, Germany) for 28 days via the water (6 l in experiment 1 and 5 l in experiment 2) in separate plastic tanks (Ferplast, Vicenza, Italy). In experiment 1, the exposure groups were: solvent control, 1, 10, 100 ng NET l^−1^, and 10 and 100 ng P l^−1^ (nominal concentrations), *n* = 6 tanks per exposure group with one individual female per tank. In experiment 2, the exposure groups were: solvent control, 1, and 10 ng NET l^−1^ and 1 and 10 ng P l^−1^ (nominal concentrations), *n* = 10 for controls and *n* = 6 for the NET and P groups, where *n* = number of tanks, with one individual female per tank. The concentration of the solvent acetone was 0.0002% in all tanks including the controls. The exposure was carried out under semi-static conditions, with half the test solution and water being renewed three times a week. Water samples were collected for chemical analysis once a week, before and after water change. To ensure that all females were in the same reproductive state at the start of the exposure, human chorionic gonadotropin (Sigma-Aldrich, St. Louis, MO, USA) was injected into the dorsal lymph sac which caused all females to ovulate prior to the experiment.

The frogs were maintained in a 12 L : 12 D cycle. Temperature, pH and conductivity were recorded and nitrite and ammonium levels were measured using standard tests from Sera (Gibbon, Sweden). The frogs were fed Horizon XP23 pellets (Skretting, Stockholm, Sweden) or Frog & Tadpole Bites (HBH, Springville, UT, USA) three times a week. There were no indications of exposure-related mortality, weight loss or other signs of general toxicity. The overall survival rate was 83%. Detailed information on health status and water quality is found in the electronic supplementary material, under ‘Health status and water quality’ and in the electronic supplementary material table S1.

#### (ii) Sampling and data analysis

A sample (1–2 g) was excised from the ovary (always from the same region) and fixed in formaldehyde. The tissue was dehydrated, embedded in hydroxyethyl methacrylate, sectioned and stained with haematoxylin–eosin as described in Rasar & Hammes [41]. Our previous data on the distribution of oocyte stages in *X. tropicalis* [34] have shown that there is a low variability between histological sections from various parts of the ovary as well as between individuals in the same exposure group. In fact, the distribution of oocyte stages in the control animals in our previous work [34] corresponded well with what is normal in this phylum (reviewed in [42]). Therefore, in one random ovarian section per individual all the oocytes (approx. 300) were scored as previtellogenic, vitellogenic, mature postvitellogenic or atretic according to the criteria in Hausen & Riebesell [43] using a light microscope at 200× magnification. Previtellogenic oocytes (stage I–II) have not incorporated vitellogenin, and vitellogenic oocytes (stage III–V) are undergoing vitellogenesis (vitellogenin granules are clearly visible close to the follicle cells from the beginning of stage III and display an increasingly uniform distribution in the cytoplasm at later stages). The postvitellogenic oocytes are mature, containing an unpigmented equatorial belt separating the animal and vegetal hemisphere. Oocytes undergoing degeneration (containing areas lacking staining) were scored as atretic. The proportions of various stages of follicular oocytes were calculated as percentages of the total number of follicular oocytes per section.

All histological evaluations of oocyte frequencies were made using coded slides. In experiment 1, all evaluations were made by one person only. In experiment 2, two persons evaluated all slides independently and a mean value was calculated. The distribution of oocyte stages in the control ovaries were consistent with that previously reported for *X. tropicalis* [34] and *X. laevis* [42]. Two exposure experiments were run to evaluate the reproducibility of the results.

#### (iii) Statistical analysis

The statistical analysis of the biological and water quality data was performed in GraphPad Prism v. 5.0 (GraphPad Software, San Diego, CA, USA) using the one-way analysis of variance (ANOVA) if the data passed the Kolmogorov–Smirnov normality test, otherwise the Kruskal–Wallis test followed by Dunn's multiple comparison test were used. The *F*-test of equal variance showed no differences between the exposure groups. The level of significance was 0.05.

### (b) Results

#### (i) Ovarian histology

The results show that both NET and P caused an increased proportion of previtellogenic oocytes and a decreased proportion of vitellogenic oocytes, implying inhibited vitellogenesis (table 1). The effects were ascertained at 1 ng NET l^−1^ (the lowest tested nominal concentration) and at 10 ng P l^−1^ (nominal) in two independent experiments, demonstrating reproducibility of the results. The mechanism for the inhibitory action of NET and P on the production of vitellogenic oocytes in amphibians is currently not understood. Progesterone has been shown to inhibit the uptake of vitellogenin into the amphibian oocyte *in vitro* [44] and to reduce vitellogenin synthesis in lizards, birds and fish [45–47], suggesting that both these mechanisms may be involved.

**Table 1.** Frequencies of oocyte stages (mean (s.d.)) in ovaries of female *X. tropicalis* after exposure to progesterone (P) or norethindrone (NET). Statistical significance in probability tests is indicated by asterisks.

treatment (ng l^−1^)	immature oocytes^a^ (%)	follicular oocyte stages^b^ (%)			
		previtellogenic oocytes	vitellogenic oocytes	postvitellogenic mature oocytes	atretic oocytes
experiment 1					
control (*n* = 5)	5.4 (3.3)	55 (6)	34 (7)	11 (3)	0.3 (0.6)
NET 1 (*n* = 4)	30 (36)	71 (6)*	21 (4)*	7 (6)	0.6 (0.2)
NET 10 (*n* = 6)	36 (42)	62 (12)	27 (3)	11 (9)	0.3 (0.5)
NET 100 (*n* = 4)	42 (47)	70 (9)	21 (7)*	9 (6)	0.4 (0.4)
P 10 (*n* = 5)	7.3 (3.6)	79 (14)**	16 (11)**	4 (3)*	0.4 (0.1)
P 100 (*n* = 6)	45 (37)*	70^c^ (5)*	24^c^ (4.45)	6 (4)^c^	0.5^c^ (0.4)
experiment 2					
control (*n* = 8)	3.3 (2.5)	58 (12)	34 (10)	6 (3)	1.9 (1.4)
NET 1 (*n* = 5)	3.6 (0.9)	78 (9)*	18 (7)*	4 (1)	1.0 (1.5)
NET 10 (*n* = 4)	2.7 (0.8)	79 (9)*	18 (7)*	3 (1)	0.9 (0.8)
P 1 (*n* = 5)	2.8 (2.2)	71 (6)	23 (5)	4 (2)	1.7 (0.8)
P 10 (*n* = 6)	4.0 (3.5)	77 (14)*	17 (10)**	4 (3)	1.7 (1.4)

^a^Percentage of oocytes in early meiotic prophase of the estimated total number of oocytes in a histological section of the ovary.

^b^Percentage of follicular stage oocytes of the total number of follicular oocytes in a histological section of the ovary.

^c^*n* = 5, one individual in the P 100 ng l^−1^ group had extremely few follicular oocytes (0.2%) and was therefore omitted from the statistical analysis.

*p < 0.05, **p < 0.01.

#### (ii) Chemical analysis

The chemical analysis showed that, in spite of the biological effects observed in two repeated experiments, the concentrations of NET and P were below detection limit in the water samples from the exposure tanks. The limit of quantification (LOQ) for NET and P was 0.5 ng l^−1^. Notably, the measured NET and P concentrations in the stock solutions were very close to the nominal concentration of 500 mg l^−1^, 501 ± 18 mg NET l^−1^ (*n* = 6) and 505 ± 22 mg P l^−1^ (*n* = 6). Consequently, the actual concentrations of NET and P in the exposure tanks were most probably similar to or lower than the nominal concentrations, not higher. In stability tests of NET or P using the same plastic tanks as those used for the exposure experiments no degradation of the compounds was seen over 28 days. On day 28 the concentrations were determined to be 104 ± 13 ng l^−1^ of NET and 98 ± 12 ng l^−1^ of P (*n* = 3), versus nominals of 100 ng l^−1^, i.e. less than 5% was lost. Addition of frog feed into the water was shown not to influence the concentrations of NET and P. The measured levels were 102 ± 11 ng l^−1^ of NET and 103 ± 14 ng l^−1^ of P (*n* = 3) 48 after the addition of the feed. Data from the stability trials are presented in the electronic supplementary material. The results of the stability tests further strengthen the assumption that the test concentrations in the exposure tanks were most probably similar to the nominal concentrations. The reason for the lack of measurable levels in the water samples from the exposure tanks is currently not understood but was most probably due to degradation and sorption in the containers used for medium to long-term storage (5–24 months) of the water samples prior to analysis.

### (c) Discussion

When determining environmental risks of pharmaceuticals, consideration should be given to potential additive effects of compounds that share a mode of action. Oestrogenic chemicals have been demonstrated to have additive effects at environmentally relevant concentrations highlighting the risk for underestimating the hazard and, therefore, the risk posed by real-life mixtures of chemicals that act via a similar mode of action [48]. Concentrations of individual steroids in surface waters are often in the low ng l^−1^ range or lower. Consequently, at sites where several compounds of the same type, for example, different progestagens, are present simultaneously the total concentration will probably be at least 1–10 ng l^−1^ [10,11,14,16].

Developmental exposure to LNG was shown to target Müllerian duct differentiation and oocyte development in female frogs, whereas reproductive organ development in males was less sensitive [33]. The ability of progestogens to induce developmental effects at environmentally relevant concentrations remains to be investigated. The present and previous findings show that the three progestagens, LNG, NET and P, inhibit oogenesis in the same manner, i.e. by interrupting formation of vitellogenic oocytes, after exposure of adult frogs to low ng l^−1^ concentrations. Suppressed vitellogenesis is associated with reduced egg production and therefore reproductive success, which has been demonstrated in fish [49]. Hence, the present and previous findings imply that several progestagens (LNG, NET and P) impair reproductive function in amphibians at environmental concentrations. Further investigation to elucidate whether these three progestagens can act additively to harm egg development in amphibians is warranted.

### (d) Conclusion

A number of hormonally active pharmaceuticals including steroid hormones, SSRIs and aromatase inhibitors are present at low ng l^−1^ levels in amphibian breeding habitats. In this paper, literature data on the toxicity of hormonally active pharmaceuticals in amphibians were reviewed and it was found that very little data were available regarding adverse effects of environmentally relevant exposure concentrations. EE_2_ and the progestagens LNG, NET and P have been shown to impair reproductive functions at environmentally relevant exposure concentrations, which may indicate a risk to wild amphibians. The progestagens are of particular concern given their prevalence, the range of compounds and that several of them (LNG, NET and P) share the same target—oogenesis—at environmental exposure concentrations, indicating a risk of adverse effects on fertility in wild amphibians.

## Supplementary Material

Supplementary material
