# Supplementary material for: Risks of hormonally active pharmaceuticals to amphibians: a growing concern regarding progestagens
Source: Philos Trans R Soc Lond B Biol Sci. 2014 Nov 19;369(1656):20130577. doi: 10.1098/rstb.2013.0577 (PMC4213589; doi:10.1098/rstb.2013.0577)
Supplement: Supplementary material [file rstb20130577supp1.pdf]

Säfholm, M. *et al.* 2014. Risks of hormonally active pharmaceuticals to amphibians: a growing concern regarding progestagens. *Phil. Trans. R. Soc. B.* **369** doi: 10.1098/rstb.2013.0577

Supplementary Material

Health status and water quality

The overall survival rate in the two experiments was 83 % (81 % in the control groups, 75, 83, and 67 in NET 1, NET 10, NET 100 respectively, and 83, 92 and 100% in P 1, P 10 and P 100 respectively). There were no indications of exposure related mortality, weight loss or other signs of general toxicity. No differences in water quality were found between the exposure tanks. The condition of the water in the test tanks were kept stable during the exposures with a temperature of 26± 0.5 ºC, a pH at 8 ±0.5 and oxygen saturation at 64±5%. The conductivity was 130-150 µS/cm in *experiment 1* and 470-490 µS/cm in *experiment 2*. The overall mean (S.D.) levels of ammonia (mg/L) were 0.05± 0.15 in *experiment 1* and 0.04 ± 0.03 in *experiment 2*, and the mean nitrite levels (mg/L) were 0.22± 0.0004 in *experiment 1* and 2.8± 0.8 in *experiment 2* (Table S1).

Chemical analysis

Actual NET and P concentrations in the stock solutions and tank water were analyzed using an in-line SPE column coupled to liquid chromatography-tandem mass spectrometry. In short, a triple stage quadruple MS/MS TSQ Quantum Ultra EMR (Thermo Fisher Scientific, San Jose, CA, USA) coupled with an Accela and a Surveyor LC pump (Thermo Fisher Scientific, San Jose, CA, USA) and a PAL HTC autosampler (CTC Analytics AG, Zwingen, Switzerland) were used as analytical system. Labeled ethinylestradiol ($^{13}C_2$-EE2) and LNG (D_6-LNG) were used as surrogate standards and the SRM data used were: NET; tube lens 85, 299.0 → 109.0, collision energy 24V; 299.0→91.3, collision energy 35V; P; tube lens 85, 315.1 → 109.3, collision energy 24V; 315.1→97.3, collision energy 20V; D6- LNG; tube lens 101, 319.1 → 251.3, collision energy 20V; 319.1→131.1, collision energy 34V; 13C2-EE2; tube lens 58, 281.1 → 159.1, collision energy 22V. Mass transition was set to 20% as criterion for positive identification. The limit of quantification (LOQ) for NET and P was 0.5 ng/L. The LOQ was determined as the lowest concentration within the linear range of the calibration curve for each analyte. LOQs for the tank water samples were calculated based on the instrument LOQs and scores based on matrix effects assessments. Precision tests,

including the precision of extraction and the instrumental response, were conducted by performing multiple injections (n=7) of a 100 ng/L calibration standard. Individual stock solutions of NET and P were prepared in methanol and stored at -18 C. Milli-Q water was injected after certain calibration points to assess the impact of memory effects on the analytical results. In order to evaluate the method's linearity, a seven point calibration curve was prepared for analyte concentrations ranging from 0.5 ng/L to 500 ng/L. The water samples taken from the exposure tanks and stock solutions were stored frozen (-18 ºC) for 5-8 months (experiment 2) and up to 2 years (experiment 1) prior to processing for analysis.

Chemical stability

All stock solutions (500 mg/L stored in acetone) were analyzed and the levels of NET and P were 501±18 mg/L (n=6) and 505 ±22 mg/L (n=6), respectively. Stability experiments during 28 days were performed in identical plastic tanks as those used in the exposure experiments. Water was spiked with 102 ±3.6 ng/L of NET and 105 ±6.1 ng/L of P (n=3). All settings were kept the same as in the exposure experiments, i.e. the average temperature was 26±0.7 ºC, pH was 7.9 ±0.4. After 28 days the concentrations were determined to be 104 ±13 ng/L of NET and 98 ±12 ng/L of P (n=3), i.e. less than 5% was lost. Another stability experiment in which frog feed (Horizon XP23 pellets (Skretting, Stockholm, Sweden) used in the exposure experiment was added to the water (1 g/L) did not show any additional loss of NET or P. The measured levels were 102 ±11 ng/L of NET and 103 ±14 ng/L of P (n=3) after 48 hours of exposure.

Table S1. Mean concentrations (S.D.) of ammonia and nitrite in the exposure tanks over the 4-week exposure period. n refers to the number of tanks.

Experiment 1

Treatment (ng/L)	Ammonia (mg/L)	Nitrite (mg/L)
Control (n=5)	0.01 (0.01)	0.20 (0.25)
NET 1 (n=4)	0.10 (0.18)	0.27 (0.24)
NET 10 (n=6)	0.06 (0.22)	0.23 (0.26)
NET 100 (n=4)	0.01 (0.01)	0.23 (0.25)
P 10 (n=5)	0.01 (0.01)	0.22 (0.26)
P 100 (n=6)	0.12 (0.20)	0.19 (0.25)

Experiment 2

Exposure concentration (ng/L)	Ammonia (mg/L)	Nitrite (mg/L)
Control (n=8)	0.04 (0.01)	2.9 (5.7)
NET 1 (n=5)	0.05 (0.06)	2.7 (4.8)
NET 10 (n=4)	0.04 (0.02)	2.0 (4.8)
P 1 (n=5)	0.04 (0.02)	2.4 (5.4)
P 10 (n=6)	0.04 (0.03)	4.1 (7.4)
